# How Does Reprogramming to Pluripotency Affect Genomic Imprinting?

**DOI:** 10.3389/fcell.2019.00076

**Published:** 2019-05-09

**Authors:** Valentina Perrera, Graziano Martello

**Affiliations:** Department of Molecular Medicine, School of Medicine and Surgery, University of Padova, Padua, Italy

**Keywords:** imprinting, reprogramming, IPS, epigenetics, pluripotency, stem cells

## Abstract

Human induced Pluripotent Stem Cells (hiPSCs) have the capacity to generate a wide range of somatic cells, thus representing an ideal tool for regenerative medicine. Patient-derived hiPSCs are also used for *in vitro* disease modeling and drug screenings. Several studies focused on the identification of DNA mutations generated, or selected, during the derivation of hiPSCs, some of which are known to drive cancer formation. Avoiding such stable genomic aberrations is paramount for successful use of hiPSCs, but it is equally important to ensure that their epigenetic information is correct, given the critical role of epigenetics in transcriptional regulation and its involvement in a plethora of pathologic conditions. In this review we will focus on genomic imprinting, a prototypical epigenetic mechanism whereby a gene is expressed in a parent-of-origin specific manner, thanks to the differential methylation of specific DNA sequences. Conventional hiPSCs are thought to be in a pluripotent state primed for differentiation. They display a hypermethylated genome with an unexpected loss of DNA methylation at imprinted loci. Several groups recently reported the generation of hiPSCs in a more primitive developmental stage, called naïve pluripotency. Naïve hiPSCs share several features with early human embryos, such as a global genome hypomethylation, which is also accompanied by a widespread loss of DNA methylation at imprinted loci. Given that loss of imprinting has been observed in genetic developmental disorders as well as in a wide range of cancers, it is fundamental to make sure that hiPSCs do not show such epigenetic aberrations. We will discuss what specific imprinted genes, associated with human pathologies, have been found commonly misregulated in hiPSCs and suggest strategies to effectively detect and avoid such undesirable epigenetic abnormalities.

## hiPSCs and Genetic Mutations

Human induced pluripotent stem cells (hiPSCs) display the important properties of long-term self-renewal and pluripotency: they are theoretically capable of generating unlimited amounts of any differentiated cell of the human body (Takahashi et al., 2007; Yu et al., 2007). For these reasons, hiPSCs represent a valuable tool for regenerative medicine, thus their safety has to be proven, particularly in terms of genetic and epigenetic stability. A significant number of large and point mutations has been reported in all hiPSC genome-wide studies to date, raising considerable concerns over their safety for clinical applications (Ben-David and Benvenisty, 2011; Turinetto et al., 2017; D’Antonio et al., 2018). For instance, the first clinical trial that used autologous hiPSCs was suspended because three single nucleotide variations (SNVs) and three copy-number variations (CNVs) were detected in hiPSCs, that were not detectable in the patient’s original fibroblasts (Garber, 2015; Blair and Barker, 2016). One of the SNVs identified is listed in a curated database of somatic cancer-associated mutations, although only linked to a single cancer (Garber, 2015). Single nucleotide variations or polymorphisms (SNVs or SNPs) are frequently observed in the population. SNVs are often found in intergenic regions, thus creating no harm, but they can also occur within the coding portion of a gene, potentially generating a mutated or truncated protein. CNVs are a type of structural variation involving alterations in the number of copies of specific regions of DNA, which can either be deleted or duplicated. These chromosomal deletions and duplications involve quite large stretches of DNA, which may span many different genes, causing potentially dangerous mutations.

**FIGURE 1:**
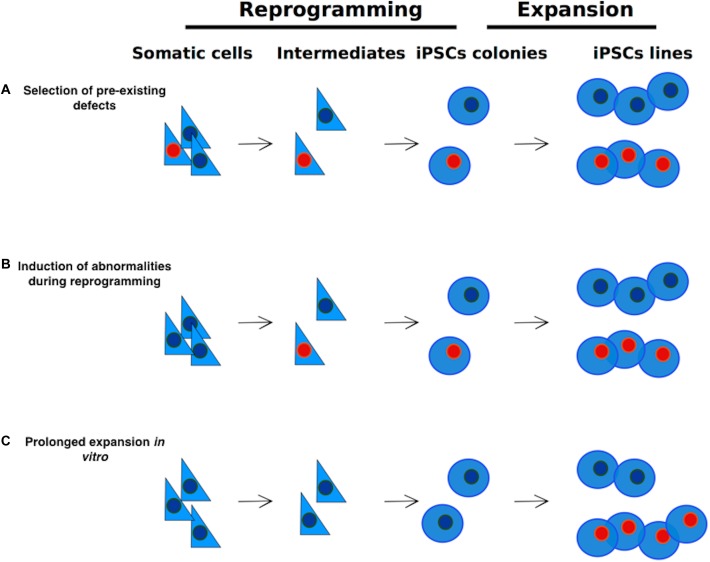
Sources of genetic alterations in iPSCs. Genetic alterations in iPSCs may arise via three different routes: **(A)** selection and expansion of abnormalities that are already present in somatic cells; **(B)** reprogramming process-induced alterations; **(C)** Alterations induced by extensive culture. Cells carrying abnormalities are indicated in the figure with a red nucleus.

There are three possible sources of genetic alterations in iPSCs (Figure 1; see also Liang and Zhang, 2012; Yoshihara et al., 2017):

(a) During the reprogramming process some pre-existing abnormalities, such as somatic mutations may be selected and expanded;
(b) The reprogramming process might generate rare alterations *per se*;
(c) *In vitro* expansion of hiPSCs for extended passaging might cause the generation of advantageous mutations.

### Genetic Mutations in hiPSCs: Selection and Expansion of Pre-existing Abnormalities

An extensive study conducted on hiPSCs generated from different types of donor cells found a similar mutation rate for both coding and non-coding regions, arguing against a functional role for such mutations (Ruiz et al., 2013). Ruiz and colleagues also showed that mutations are not occurring preferentially in expressed genes, but they rather spread throughout both transcriptionally active and silent regions of the genome. Most of the mutated genes mapped in the study did not facilitate reprogramming through a gain-of-function or loss-of-function mechanism and much of the genetic variation in hiPSC clones pre-existed in the somatic population of origin and was passively fixed as a consequence of cloning individual cells during hiPSC generation (Ruiz et al., 2013; Kwon et al., 2017). Additional studies investigated the incidence of SNVs after reprogramming, confirming that only few SNVs occur within coding regions (<10 SNVs per clone, Cheng et al., 2012; Su et al., 2013).

Sardo and colleagues measured the rate of mutations in blood cells and hiPSCs derived from them. Despite a correlation between donor age and the number of mutations observed, there was no evidence for positive selection of somatic mutations in hiPSC, with a large degree of heterogeneity in the somatic mutations identified between lines derived from the same individual (Sardo et al., 2016). A similar high variability in mutations observed in isogenic clones was also reported by others (Popp et al., 2018; Wang et al., 2019). The number of mutations was independent of the somatic cell type used for reprogramming. Older cells carry a higher number of genetic mutations than younger cells, simply because they have gone through a higher number of cell divisions and they have been exposed for a longer time to environmental mutagenic triggers. Therefore the likelihood of genetic aberrations occurrence in hiPSCs increases with the age of the donor cells to be reprogrammed. It has been recently reported that hematopoietic stem cells contain a lower load of somatic SNVs than skin fibroblasts, and such difference is maintained after reprogramming (Wang et al., 2019). Given that hematopoietic stem cells can also be reprogrammed with very high efficiency they could represent a preferred source for clinical grade hiPSCs.

In sum, genetic mutations observed in hiPSCs are in part pre-existing abnormalities of source somatic cells that are passively fixed by the process of reprogramming.

### Genetic Mutations in hiPSCs: The Reprogramming Process Induces Genetic Alterations

Induced pluripotent stem cells were originally generated using retrovirus-mediated delivery of reprogramming factors (Takahashi and Yamanaka, 2006; Takahashi et al., 2007; Yu et al., 2009), but stable integration of retroviral vectors may cause potentially dangerous mutations. In order to generate safer reprogrammed cells, alternative methods have been used, such as excisable piggyBac vectors (Woltjen et al., 2009), Sendai virus vectors (Fusaki et al., 2009), episomal plasmids (Yu et al., 2009; Okita et al., 2011) and DNA free reprogramming methods, that rely on the delivery of proteins (Kim et al., 2009; Zhou et al., 2009) or of modified messenger RNAs (mmRNAs, Warren et al., 2010; Luni et al., 2016). In particular, mmRNAs are especially attractive as they have a short half-life and are completely lost within a few cell divisions, thus allowing the generation of iPSCs free from any exogenous genetic material.

To determine whether reprogramming is associated with *de novo*-generated CNVs, human retroviral or piggyBac generated hiPSCs lines were compared to human ESCs lines and parental fibroblasts lines (Hussein et al., 2011). The study evidenced that reprogramming causes the formation of several CNVs during its early phases. Most cells bearing these CNVs are exposed to a negative selective pressure, thus resulting in the dilution of the same mutated cells over extended passaging. Several studies compared the frequency of mutations obtained with different reprogramming strategies. The expression of the Yamanaka factors or somatic nuclear transfer (SCNT – a somatic nucleus reprogrammed to pluripotency thanks to its transfer into an enucleated oocyte – Gurdon, 1962; Tachibana et al., 2013) generate a similar number of genomic aberrations (Johannesson et al., 2014; Ma et al., 2014). When different methods based on expression of Yamanaka factors are compared, the integration-free methods generate cells with slightly lower incidence of genetic variations, compared to virally transduced hiPSCs (Cheng et al., 2012; Sugiura et al., 2014; Schlaeger et al., 2015; Bhutani et al., 2016).

In sum, the process of reprogramming induces novel genomic alterations, whose incidence is reduced by the use of non-integrating methods.

### Genetic Mutations in hiPSCs: Generation of Advantageous Genetic Mutations Due to Extended Passaging

An important example of how extended culturing can induce genetic mutations is given by the reported acquisition of *P53* mutations by both hiPSCs and human Embryonic Stem Cells (hESCs) in culture. Not only cells spontaneously acquired *P53* mutations, but the fraction of cells carrying the mutant *P53* allele increased with passage number under standard culture conditions (Merkle et al., 2017).

The mutations observed mapped to the DNA-binding domain of P53, as the ones occurring in human cancers. These types of mutations often act as dominant negative and substantially diminish P53 regulation of apoptosis, cell cycle progression and genomic stability, leading to widespread DNA lesions (Merkle et al., 2017).

To conclude: pre-existing somatic mutations can be selected and expanded in clonal hiPSCs, but generally there is no evidence for a selective advantage conferred by such mutations. There is also a low rate of mutations generated during reprogramming. Finally, selection of *P53* mutations in prolonged cultures of hiPSCs and hESCs has been documented on a large number of cell lines, therefore the use of low-passage hiPSCs is preferable (see Table 1).

**Table 1:** Origin of (epi)mutations in reprogrammed pluripotent stem cells.

Mechanism	Genetic mutations (SNVs or CNVs)	Loss of imprinting in conventional (primed) hiPSCs	Loss of imprinting in naïve hiPSCs
(1) Selection of pre-existing defects	Yes	Yes	Yes
	Kwon et al., 2017	Bar et al., 2017	Bar et al., 2017
	Sardo et al., 2016	Burnett et al., 2016	
		Chamberlain et al., 2010	
(2) Induction of abnormalities during reprogramming	Yes	Yes	Not yet determined
	Johannesson et al., 2014	Ma et al., 2014	Bar et al., 2017
	Ma et al., 2014	Johannesson et al., 2014	Giulitti et al., 2019
(3) Prolonged expansion *in vitro*	Yes	Yes	Not yet determined
	Merkle et al., 2017	Stelzer et al., 2011	Bar et al., 2017
	Ruiz et al., 2013	Johannesson et al., 2014	Giulitti et al., 2019
		Hiura et al., 2013	
		Bar et al., 2017	
		Pick et al., 2009	


*There are three possible sources of genetic mutations as well as of epigenetic ones in hiPSCs. (1) Defects could exist in the somatic cells before reprogramming and then carried on to the reprogrammed cell; (2) defects could be induced by the reprogramming process; and (3) defects could be generated during the prolonged culture *in vitro*.*

## hiPSCs and Epimutations

All cells in our body contain the same genetic information, but display a different phenotype, thanks to the expression of specific groups of genes. Both the histones associated to DNA and the DNA itself can be variably modified creating a pattern of modifications that forms the so called ‘epigenetic code’ (Turner, 2007). The epigenetic code determines what genes are transcribed and, importantly, is stable over multiple cell generations, yet reversible.

The first heritable change discovered is DNA methylation (reviewed in Kim and Costello, 2017). DNA methyl marks are deposited by DNA methyltransferases on the fifth carbon of a cytosine, mostly within CpG dinucleotides [5-methylcytosine (5mC)]. 5mC is a heritable modification that represses gene expression by inhibiting the binding of transcriptional activators or by creating a site that is specifically recognized by transcriptional repressors (reviewed in Allis and Jenuwein, 2016). DNA methylation is critical for controlling gene expression, X chromosome inactivation and imprinting (reviewed in Reik and Surani, 1997; Kim and Costello, 2017).

Within the family of DNA methyltransferases, DNMT3A and 3B possess the enzymatic activity necessary for the establishment of most of the DNA methyl marks in the human genome. They are called *de novo* DNA methyltransferases, as they methylate new DNA sequences, like the imprinted genes in the gametes (Chen et al., 2003; Kaneda et al., 2004). DNA replication is semiconservative of the DNA methyl marks: from the methylated double stranded DNA, two hemimethylated double strands are generated after replication. DNMT1 functions as a maintenance DNA methyltransferase, as it recognizes newly synthesized hemimethylated CpGs and restores the fully methylated CpG dyads (Bostick et al., 2007; Sharif et al., 2007). Mouse mutants for DNA methyltransferases are embryonic lethal (Li et al., 1992; Okano et al., 1999), while genetic ablation of *DNMT1* impairs the differentiation potential of human pluripotent stem cells, thus underlining the importance of DNA methylation during early stages of development (Bogdanović and Lister, 2015; Liao et al., 2015).

While the genome sequence should be kept unaltered during reprogramming, the epigenetic modifications have to be correctly reset, such that a differentiated cell becomes pluripotent (Takahashi and Yamanaka, 2006; Takahashi et al., 2007; Papp and Plath, 2011; Brix et al., 2015; Cacchiarelli et al., 2015; Gładych et al., 2015). During this process some epimutations may be generated. The term epimutations is used to describe all kinds of aberrant modifications of the DNA or histones, that are transmitted to daughter cells. These are very relevant for the destiny of the reprogrammed cell and likely to occur if reprogramming is incomplete. As for genetic mutations, epimutations may arise via three different mechanisms that will be discussed in the following sections.

### Epimutations in hiPSCs: Pre-existing Epigenetic States Could Be Kept and Transmitted Through Cell Reprogramming

**FIGURE 2:**
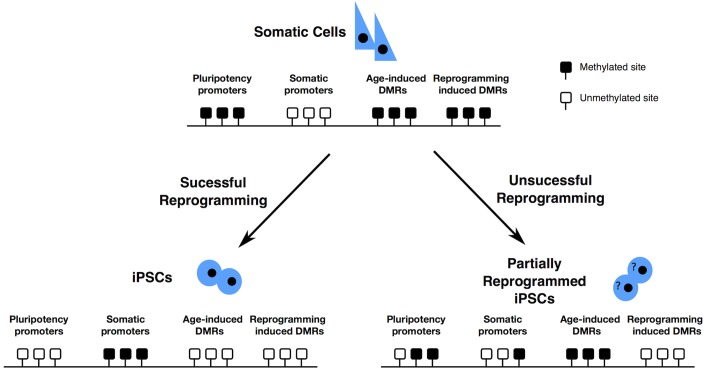
Epigenome resetting during reprogramming – Pluripotency promoters have to be demethylated, while somatic cell specific promoters have to be methylated for successful reprogramming to occur. DNA methylation accumulates at specific DMRs, with ageing. After partial reprogramming some of such DMRs are maintained. Finally, reprogramming induces the formation of specific differentially methylated regions (DMRs). Black boxes represent methylated sites; white boxes represent demethylated sites.

Reprogrammed cells could retain some chromatin marks belonging to the source cell (somatic memory), causing the insufficient silencing of lineage-specific genes or the negative regulation of pluripotency genes (Figure 2), limiting their differentiation into lineages other than the one of the source cells (Marchetto et al., 2009; Ghosh et al., 2010; Kim et al., 2010, 2011; Bar-Nur et al., 2011; Horvath, 2013, reviewed in Vaskova et al., 2013).

Older cells are more resistant to complete reprogramming (Sardo et al., 2016), probably because of the accumulation of age dependent methylation at specific loci. Aging is usually accompanied by a gradual and genome wide loss of DNA methylation (Booth and Brunet, 2016), that exposes aged individuals to a higher risk of cancer due to the consequential increased risk of accumulating genomic aberrations. Despite this, some CpG islands and gene-rich regions become hypermethylated with age. These loci displaying age-dependent DNA hypermethylation are preferentially near tissue-specific genes, genes involved in differentiation and development, genes encoding transcription factors, and transcription factor binding sites (Benayoun et al., 2015). Some of the age dependent CpGs or the somatic CpGs remain methylated even after the reprogramming process (Lister et al., 2011; Ohi et al., 2011; Sardo et al., 2016) leaving an epigenetic signature of age or of somatic origin in the hiPSCs (Figure 2). These regions are associated with alterations on histone modifications patterns and consequential difference on gene expression and are transmitted to differentiated cells at a high frequency. This phenomenon possibly generates hiPSCs with a limited differentiation potential.

### Epimutations in hiPSCs: Effects of the Reprogramming Process

The analysis of the DNA methylation profile of cells reprogrammed with different methods revealed profound differences. Somatic cell nuclear transfer (SCNT) is able to generate reprogrammed cells displaying a DNA methylation profile comparable to embryo-derived ESCs, while hiPSCs reprogrammed via the Yamanaka factors show a divergent profile, with memory of the somatic cell methylome (Figure 2) (Ma et al., 2014). A possible explanation for the more effective reprogramming by SCNT is that the ooplasm may contain specific proteins or small molecules, that together with the pluripotency factors, help the reprogramming process, making it most efficient and complete (Han et al., 2015).

The reprogramming process frequently generates aberrantly methylated regions that are dissimilar to the somatic donor cell and the human ESCs (Lister et al., 2011): these represent a class of differentially methylated regions (DMRs) that is specific to the reprogrammed cells. These aberrant DNA methylation patterns in hiPSCs often occur within CpG islands, associated with genes. A high proportion of these reprogramming specific DMRs are found in multiple independent hiPSC lines and are characterized by a reduction in DNA methylation (Lister et al., 2011).

### Epimutations in hiPSCs: Culture-Induced Epigenetic Defects

While extended culturing exposes cells to the risk of accumulation of genetic defects, prolonged expansion seems to allow the loss of the epigenetic memory, thus reducing the differences between hiPSCs and hESCs (Nishino et al., 2011). At the same time, the risk to induce epigenetic abnormalities increases with extensive passaging, as exemplified by the dynamics of X chromosome inactivation in female hiPSCs (Silva et al., 2008; Tchieu et al., 2010).

X-chromosome inactivation is the phenomenon by which transcription from one of the X chromosomes is partially silenced in female mammalian cells (Carrel and Willard, 2005; Cotton et al., 2013). Thus, X-chromosome inactivation equalizes dosage of gene expression between females and male cells. Silencing, once established, is stable: the same X chromosome remains inactivated in all subsequent cell generations, although up to 15% of X-linked genes are expressed also from the inactive X, with variability between tissues and individuals (Balaton and Brown, 2016). The stable silencing of the X chromosome is the product of multiple epigenetic mechanisms, such as DNA methylation, deposition of repressive histone marks and coating by the long non-coding RNA *XIST* (reviewed in Galupa and Heard, 2015).

During the initial phases of female hiPSCs and hESCs generation, both X chromosomes are active (Lengner et al., 2010; Kim et al., 2014), but upon passaging one X chromosome undergoes inactivation, as indicated by XIST coating. However, after prolonged culture, the inactive X chromosome loses the XIST coating and partially reactivates gene expression (Silva et al., 2008; Vallot et al., 2015). Such phenomenon, described as “erosion” of the inactive X, seems to generate cells with a proliferative advantage, probably because of the enhanced expression of oncogenes encoded by the X chromosomes (Anguera et al., 2012). These cells display a reduced differentiation potential, because the eroded X is passed to differentiated daughter cells and never undergoes X inactivation (Mekhoubad et al., 2012; Nazor et al., 2012; Kim et al., 2014; Patel et al., 2017), leading to unbalanced expression of genes on the X chromosome.

The causes of such dynamic changes of the X chromosome status are only partially understood. For instance, culture under hypoxic conditions or in the presence of the cytokine LIF (Tomoda et al., 2012) allows the activation of both X chromosomes, through some yet undefined mechanisms (see also Cantone and Fisher, 2017).

In sum, somatic cell type- or age-dependent epigenetic profiles are partially maintained during hiPS derivation with the Yamanaka factors, while SCNT is more efficient at erasing them. Extended cultures help reducing the differences between hESCs and hiPSCs in terms of epigenetic profile, but at the same time expose to the risk of other epigenetic abnormalities such as the erosion of the chromosome X.

A special type of heritable regulation of gene expression – genomic imprinting – is also affected in hiPSCs. We will focus on how reprogramming to pluripotency compromises its stability and comment on the potential consequences of loss of imprinting (LOI).

## Misregulation of Imprinting in hiPSCs

The majority of the autosomal genes are expressed from both copies; however, a small subset of genes has one copy turned off in a parent-of-origin dependent manner. These genes are called ‘imprinted.’ In this class of genes one allele is marked with DNA methylation. Imprinted genes acquire DNA methylation during gametogenesis and maintain their DNA methylation profile stable in all adult tissues (Okae et al., 2014; Plasschaert and Bartolomei, 2014; Gkountela et al., 2015). Thus, the allelic expression of an imprinted gene depends upon whether it was inherited from the mother or the father and its stability relies on the integrity of DNA methylation over some regions that are differentially methylated in the two alleles, thus serving as imprinting control regions (reviewed in Ferguson-Smith, 2011; Elhamamsy, 2017).

Imprinted genes usually form clusters, where several coding and at least one non-coding RNA are under the control of one unique DMR. These DMRs can be intergenic or intragenic. Interestingly, paternal DMRs are few in number and all intergenic, while maternal DMRs are more abundant and all reside within genes (Messerschmidt et al., 2014). Genetic deletions of DMRs cause a LOI, manifested as a loss of monoallelic expression, underlining the importance of a DMR for the imprinted gene cluster regulation (Ishida and Moore, 2013; Barlow and Bartolomei, 2014; Kalish et al., 2014; Moore et al., 2015).

The loss or gain of DNA methylation over DMRs can both result in the monoallelic to biallelic conversion of gene expression or in the repression of the only transcriptionally active allele. This leads to alterations in the dosage of the imprinted transcripts that are potentially harmful. Indeed, the monoallelic expression of imprinted genes allows a tight control of their dosage and is essential for the proper development of the embryo (Ishida and Moore, 2013).

Loss of imprinting, particularly at a small subset of imprinted genes. The imprinted genes most commonly found biallelically expressed in conventional hiPSCs are *H19, IGF2, MEG3, PEG3, PEG10, MEST* (Rugg-Gunn et al., 2005, 2007; Kim et al., 2007; Pick et al., 2009; Hiura et al., 2013; Johannesson et al., 2014; Ma et al., 2014; Bar et al., 2017). Interestingly, paternal DMRs seem more affected by LOI than maternal ones (Rugg-Gunn et al., 2007; Bar et al., 2017), indicating that in pluripotent stem cells two different mechanisms are in place to maintain the two classes of imprinted genes.

The same three principles that we described for the genetic mutations (Figure 1) in hiPSCs apply to epimutations, such as LOI. These are summarized in Table 1.

### Loss of Imprinting in Conventional hiPSCs: Pre-existing Aberrant Imprinting States Could Be Kept and Transmitted Through Cell Reprogramming

Similarly to the pre-existing somatic genetic mutations in the donor cells, that would be carried to the reprogrammed cells, LOI is sometimes already evident in the somatic cells used for reprogramming (Bar et al., 2017).

Aberrant imprints present in the somatic cell of origin are mostly faithfully retained in hiPSCs after reprogramming, as shown by the maintenance of the abnormal state of imprinting in hiPSCs generated from fibroblasts of Angelman or Prader Willi syndromes patients, two well-known imprinting disorders (Chamberlain et al., 2010; Burnett et al., 2016).

### Loss of Imprinting in Conventional hiPSCs: Reprogramming -Induced Imprinting Instability

Reprogrammed pluripotent stem cells (either hiPSCs or SCNT-pluripotent stem cells) are more susceptible to LOI than hESCs (Pick et al., 2009; Johannesson et al., 2014; Bar et al., 2017): this suggests that the reprogramming process has a negative impact on the stability of imprints. Furthermore, different reprogramming techniques cause a different degree of LOI: Ma et al. (2014) reported a more pronounced aberrant DNA methylation at imprinted loci in hiPSCs reprogrammed with the Yamanaka factors compared to SCNT-derived cells. Such results suggest that the ooplasm might contain molecules critical for the correct resetting of the epigenetic profile of the nucleus.

### Loss of Imprinting in Conventional hiPSCs: Culture-Induced Imprinting Defects

During normal development imprinting is very stable, but experimental manipulations of pluripotent cells are known to affect it. For instance, Assisted Reproductive Technologies that rely on the manipulation of oocytes and embryos *in vitro*, have been shown to induce a significant increase in the occurrence of imprinting disorders like Beckwith–Wiedemann syndrome in babies conceived using such techniques (reviewed in Uyar and Seli, 2014). This indicates that *in vitro* culture may trigger LOI in gametes and pluripotent cells of the embryo. Imprinted loci show a certain instability during culture of pluripotent stem cells: LOI has been demonstrated at some imprinted loci in hESCs or hiPSCs kept in culture (Bar et al., 2017), similarly to the erosion of X chromosome inactivation.

Additionally, given that some imprinted genes are regulators of growth, it is possible that the loss of their imprinted status might occur during expansion *in vitro*, because of the advantage conferred to the cells. This might explain why imprinting is lost on some of them at a higher frequency than on others. One such example is *IGF2*, a promoter of proliferation that is associated with many types of cancer (Morison and Reeve, 1998; Cui et al., 2002, 2003; Kaneda and Feinberg, 2005; Rugg-Gunn et al., 2007) and with the overgrowth phenotype observed in Beckwith–Wiedemann syndrome (Tatton-Brown et al., 2013).

## Misregulation of Imprinting in Human Naïve hiPSCs

Murine pluripotent stem cells differ from conventional hiPSCs, as the latter are thought to be in a more advanced developmental stage called primed state of pluripotency. In the embryo, naïve pluripotent cells are found in the inner cell mass of the pre-implantation blastocyst. Such cells undergo morphological and transcriptional rearrangements in preparation for germ layer formation, while retaining pluripotency. Thus, pluripotent cells found in the embryo after implantation are considered in a pluripotent state primed for differentiation.

The difference between the primed state and the naïve state is evident at the metabolic, transcriptional, and epigenetic levels (Nichols and Smith, 2009; Davidson et al., 2015; Weinberger et al., 2016; Takahashi et al., 2017; Kilens et al., 2018).

Recent establishment of alternative culture conditions has allowed the derivation and maintenance of human cells in a naïve pluripotent state (Davidson et al., 2015; Weinberger et al., 2016; Takahashi et al., 2017). Naïve pluripotent stem cells are characterized by a hypomethylated genome (Leitch et al., 2013; Pastor et al., 2016; Theunissen et al., 2016; Liu et al., 2017; Wang et al., 2018). This state, that is only transient *in vivo*, can be reproduced indefinitely *in vitro*, which might lead to abnormalities. Indeed, naïve cells display also a decrease or even a complete loss of methylation at imprinted DMRs (Liu et al., 2017). This loss of DNA methylation results in some cases in the biallelic expression of the genes controlled by the DMR (Pastor et al., 2016; Bar et al., 2017; Giulitti et al., 2019). Moreover, DNA methylation, when severely lost at an imprinted DMR is not restored upon conversion from naïve to primed cells (Pastor et al., 2016) or after differentiation (Bar et al., 2017).

Such findings could be explained in light of the known mechanisms for the maintenance of DNA methylation at imprinted loci. During the wave of demethylation that occurs in the early embryo, the KAP1 co-repressor complex recruits DNMT3A and DNMT3B to imprinted genes, thanks to the interaction with ZFP57 that binds to a methylated CG within an hexanucleotidic recognition motif (TGCCGC) found within the imprinted DMRs (reviewed in Quenneville et al., 2011; Zuo et al., 2012; Baglivo et al., 2013; Messerschmidt et al., 2014; Strogantsev et al., 2015). Of note, mutations at *ZFP57* gene are associated with imprinting disorders, such as transient neonatal diabetes (Mackay et al., 2008). If the binding motif of ZFP57 is completely demethylated, ZFP57 will not recognize it and no recruitment of KAP1 and DNMTs will occur to maintain DNA methylation or to re-establish the lost methylation (Zuo et al., 2012; Voon and Gibbons, 2016). It would be interesting to test whether binding of ZFP57, KAP1, and DNMTs at imprinted loci is lost in naïve cells. Moreover, a more extensive study based on global transcription analysis is needed to establish the mono- versus bi-allelic expression pattern of imprinted genes in human naïve ESCs in culture. This should give a more direct measurement of the functional loss of imprinted transcription that in some cases could be maintained also in the absence of DNA methylation.

The loss of imprinting observed in naïve hiPSCs could be the combined effect of:

(1) Expansion of pre-existing LOI: in Giulitti et al. (2019), some DMRs (IG-DMR, NHP2L1, ZNF331_1, HTR5A, FAM50B, WRB, DIRAS3) display a low DNA methylation already in some of fibroblast lines. These low levels of DNA methylation are either maintained low or even reduced in the matching naïve hiPSCs.
(2) The reprogramming process itself: as observed in primed hiPSCs, the process of reprogramming could generate imprinting abnormalities *per se.* In order to evaluate such hypothesis, low passage naïve hiPSCs should be compared to matching fibroblasts, to see if the DNA methylation profile of imprinted genes has undergone any change during the reprogramming process and consequently if their monoallelic expression has been maintained or lost.
(3) A result of the extensive passaging, cell expansion and selection: over several rounds of cell divisions, a cell with global low levels of DNA methylation could easily undergo LOI. To investigate this, low- and high-passage naïve cells should be compared to see if there is any difference in the levels of DNA methylation and if some imprinted genes acquired biallelic expression.

Although additional analyses will be needed to tease out the contribution of the three possible mechanisms listed above, we should also discuss studies performed in mouse naïve pluripotent stem cells. When mouse naïve pluripotent stem cells are expanded in serum-free medium containing two inhibitors of the MEK and GSK3 kinases and the cytokine LIF (2i+LIF), they show a hypomethylated genome, as also reported for pluripotent cells of the pre-implantation embryo (Messerschmidt et al., 2014). In contrast, mouse naïve pluripotent stem cells cultured in serum-based medium and LIF (Serum+LIF, Ficz et al., 2013) show high levels of DNA methylation.

Even though global DNA methylation levels substantially decrease over a relatively short time when cells are passed from Serum+LIF to 2i+LIF cultures, imprints are rather stable in the same time frame and upon extended passaging (Ficz et al., 2013). This holds true only in male cells, while female cells tend to lose DNA methylation at imprinted loci (Habibi et al., 2013; Hackett et al., 2013). However, two groups reported more recently a faster and severe reduction of DNA methylation over DMRs in male and female cells cultivated in 2i+LIF (Choi et al., 2017; Yagi et al., 2017). By simply reducing the concentration of the MEK inhibitor, even female murine cells maintained stable imprints during extensive culture (Yagi et al., 2017), indicating that slight variations in the culture conditions used could have a major effect on the maintenance of DNA methylation. A recent study on naïve hESCs also showed that the reduction of the MEK inhibitor concentration was beneficial, in terms of genomic stability. However, the genome appeared hypomethylated and imprinted control regions were still completely demethylated (Di Stefano et al., 2018).

In sum, imprinting at methylated loci is more labile in female murine cells than male cells, but we do not know if the same holds true in human cells. Moreover, extensive culture leads to LOI and optimization of the media composition could improve imprinting stability also in human cells.

## Potential Hazards Linked to the Use of hiPSCs in Clinical Applications: Loss of Imprinting Is Associated to Developmental Disorders and Cancer

### Loss of Imprinting and Developmental Diseases

Imprinted genes are dosage-sensitive: their deregulated expression leads to different pathologies, ranging from cancer to developmental diseases known also as imprinting disorders (Ishida and Moore, 2013; Kalish et al., 2014).

In this section we summarize the pathologies linked to imprinted genes, focusing on those genes more often found misregulated in hiPSCS. Before hiPSCs are used for cell replacement, we should be aware of how LOI could compromise the function of specific cell types. Imprinted genes are organized in clusters under the control of single DMRs, therefore changes in DNA methylation of a DMR will result in altered expression of multiple imprinted genes (Barlow and Bartolomei, 2014). The majority of the imprinting disorders cannot be explained by absence or upregulation of a single gene product, but rather by the altered levels of expression of multiple genes in the relevant region, explaining why most imprinting disorders are spectrum disorders.

In humans, six imprinted clusters have been consistently associated with disease:

(1) IC1 (H19/IGF2)
(2) IC2 (KvDMR)
(3) DLK1/MEG3
(4) SNURF/SNRPN
(5) GNAS
*(6) PLAGL1/HYMAI*.

**FIGURE 3:**
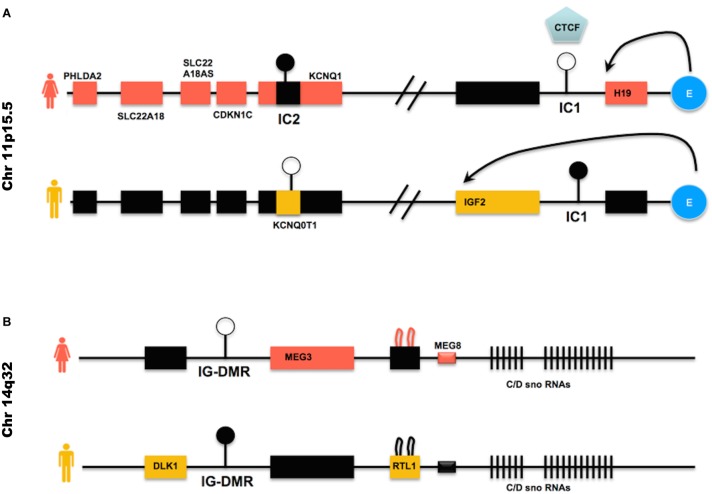
Imprinted genes clusters often deregulated in hiPSCs – **(A)** IC1 is the DMR within the *H19/IGF2* locus; the blue round shaped E represents the enhancer found within the same locus. CTCF is an insulator protein that binds to the IC1 DMR when this is not methylated (female allele), limiting the influence of the enhancer to the *H19* gene only. When the IC1 DMR is methylated (male allele), CTCF cannot bind to it and the enhancer can direct the expression of IGF2, while H19 is repressed by the methylated IC1 DMR upstream to its promoter. IC2 is the DMR found within the *KCNQ10T1/CDKN1C* cluster. When IC2 is methylated (female allele), the non-coding RNA KCNQ10T1 is repressed and KCNQ1 is expressed, as well as CDKN1C and the other genes on the female allele. When, on the male allele, IC2 is not methylated, the non-coding RNA KCNQ10T1 is expressed and this causes the repression *in cis* of all other genes described in the cluster. **(B)** IG-DMR is the DMR within the *DLK1/MEG3* cluster. IG-DMR is demethylated on the female allele, allowing the expression *of MEG3. MEG3* expression causes the repression of genes such as *DLK1*. When IG-DMR is methylated on the male allele, *MEG3* is not expressed and *DLK1* is expressed. Black boxes indicate transcriptionally silent genes. Pink or orange boxes indicate genes transcribed in female or male cells, respectively.

The imprinted genes that most frequently undergo LOI in hiPSCs either in their primed or naïve state are *H19, IGF2, KCNQ10T1, MEG3* (reviewed in Rugg-Gunn et al., 2005; Kim et al., 2007; Pick et al., 2009; Lund et al., 2012; Hiura et al., 2013; Johannesson et al., 2014; Ma et al., 2014; Bar et al., 2017; Giulitti et al., 2019) (clusters described in Figure 3).

Their deregulated expression is the cause of Beckwith–Wiedemann syndrome (H19, IGF2, KCNQ1OT1), Russel-Silver syndrome (H19, IGF2, KCNQ1OT1) and Uniparental disomy 14 (MEG3) and is also observed in several tumors (see Table 2).

**Table 2:** Imprinting disorders linked to the imprinted genes described in Figure 3 (*IGF2, H19 KCNQ10T1, CDKN1C MEG3 DLK*).

DMR	Associated transcripts showing LOI in hiPSCs	Type of defect	Disease	References
IC1	*H19*	Loss of methylation causes *H19* activation	Beckwith–Wiedemann syndrome	Azzi et al., 2014
		(BWS)	Russell-Silver syndrome	Kalish et al., 2014
		Gain of methylation causes *H19* repression (RSS)		Butler, 2009
				Elhamamsy, 2017
				Catchpoole et al., 1997
				Reik et al., 1995
				Bartholdi et al., 2009
IC1	IGF2	Loss of methylation causes IGF2 repression (BWS)	Beckwith–Wiedemann syndrome,	Azzi et al., 2014
		Gain of methylation causes IGF2 activation (RSS)	Russell-Silver syndrome	Kalish et al., 2014
				Elhamamsy, 2017
				Catchpoole et al., 1997
				Reik et al., 1995
				Bartholdi et al., 2009
IC2	KCNQ10T1, CDKN1C	Loss of methylation causes KCNQ10T1 activation and CDKN1C repression	Beckwith–Wiedemann syndrome	Azzi et al., 2014
				Kalish et al., 2014
				Gaston et al., 2001
				Algar et al., 1999
				Weksberg et al., 2002
IG DMR	MEG3, DLK1	Loss of methylation causes MEG3 activation and DLK1 repression	Uniparental disomy 14 (UPD14)	Ogata and Kagami, 2008
		Gain of methylation causes MEG3 silencing and DLK1 activation		Elhamamsy, 2017
				Sutton and Shaffer, 2000
				Kalish et al., 2014


*Listed in the table are the DMRs affected in the imprinting disorders described, the genes that most frequently encounter LOI in hiPSCs, associated to the DMRs described, the types of defects observed in the imprinting disorders, the names of the associated imprinting disorders and the references.*

### Beckwith–Wiedemann Syndrome (BWS), Russell-Silver Syndrome (RSS)

Despite the fact that BWS and RSS can be caused by mutations or LOI occurring in genes that belong to the same cluster, BWS and RSS show opposite phenotypes, with BWS characterized by fetal and extraembryonic overgrowth, while RSS is characterized by severe pre- and post-natal growth retardation (Butler, 2009; Tatton-Brown et al., 2013; Azzi et al., 2014; Kalish et al., 2014; Elhamamsy, 2017).

BWS is in most cases caused by loss of methylation at IC2 DMR (see Figure 3A for a description of the locus), the DMR present in the *KCNQ10T1/CDKN1C* cluster (Algar et al., 1999; Weksberg et al., 2002). This loss of methylation results in biallelic expression of the ncRNA *KCNQ10T1* and consequently *cis-*acting repression of the protein-coding genes regulated by *KCNQ10T1*. Among the genes regulated by *KCNQ10T1*, CDKN1C acts as a cell cycle inhibitor and growth restrictor.

BWS is less frequently caused by activation of *IGF2* and reduced *H19* expression (Reik et al., 1995; Catchpoole et al., 1997), although in these cases it is often accompanied by Wilms’s tumors and other cancers (Tatton-Brown et al., 2013).

Normally, the paternal IC1 DMR in the *H19/IGF2* cluster is methylated while it is demethylated on the maternal allele (see Figure 3A). Gain of methylation at IC1 leads to overexpression of the growth factor IGF2 (Chao and D’Amore, 2008) and downregulation of *H19*, which encodes a ncRNA implicated in growth suppression (mechanism described in Figure 3A), with a developmental consequence of overgrowth (BWS). Loss of methylation at IC1 on the other hand causes the opposite phenotype, that is *H19* upregulation and *IGF2* downregulation, generating a severe growth defect, observed in patients affected by RSS (Bartholdi et al., 2009; Begemann et al., 2010).

### Uniparental Disomy 14 (UPD14)

Human chromosome 14q32.2 carries a cluster of imprinted genes including paternally expressed genes such as *DLK1* and *RTL1*, and maternally expressed genes such as *GTL2* (alias, *MEG3*), *RTL1as* (*RTL1* antisense), and *MEG8* (see Figure 3B). The *DLK1-GTL2* intergenic DMR (IG-DMR) and the *GTL2*-DMR are extensively hypermethylated in the paternal allele and grossly hypomethylated in the maternal one. Deregulation of the genes within the *DLK1-MEG3* imprinted cluster on chromosome 14q32 is responsible for the distinct phenotypes observed in the patients of maternal and paternal UPD14 syndromes (Sutton and Shaffer, 2000; Ogata and Kagami, 2008), suffering from pre- and post-natal growth restriction, skeletal abnormalities, facial dysmorphism, premature puberty and obesity.

### Loss of Imprinting and Cancer

Two facts underline the importance of imprinting also for tissue homeostasis:

(1) Individuals affected by imprinting disorders are usually more prone to develop cancer.
(2) Loss of imprinting either by genetic mutations or epimutations, is a very common phenomenon seen in cancer, and it often is an early event (reviewed in Jelinic and Shaw, 2007; Uribe-Lewis et al., 2011).

Imprinted genes are developmental regulators, often promoting or restricting growth, thus their aberrant expression in adult tissues could induce cancer formation.

Indeed, some imprinted genes are tumor suppressors, such as *MEG3* (Astuti et al., 2005; Zhou et al., 2013), KCNQ10T1 (Nakano et al., 2006) and *CDKN1C* (Sato et al., 2005), others promote proliferation, like *IGF2* (Cui et al., 2003; Jelinic and Shaw, 2007; Lim and Maher, 2010; Monk, 2010, see Feinberg et al., 2006 for a more comprehensive list of cancer-associated imprinted transcripts). *H19* is a non-coding RNA of unknown function, which may have a role in both tumor formation and tumor suppression (Ulaner, 2003; Raveh et al., 2015; Yoshimura et al., 2018).

*IGF2* codes for insulin-like growth factor 2, a growth factor highly expressed in many types of tumors. Along with Wilms’ tumor, LOI of the *IGF2* gene is associated with many other types of cancer, including lung, colon, pancreatic, cardiac, hepatic, and ovarian tumors (Kaneda and Feinberg, 2005).

The *DLK1-MEG3* imprinted locus is altered in a series of primary human tumors (myelomas, Wilms tumors, neuroblastomas, gliomas, see Huang et al., 2006; Kawakami et al., 2006; Jørgensen et al., 2013) and *MEG3* gene expression is consequently lost in several tumor cell lines (Zhou et al., 2013). Multiple mechanisms contribute to the loss of *MEG3* expression in tumors, such as gene deletion, promoter hypermethylation, and hypermethylation of the intergenic DMRs. Re-expression of *MEG3* inhibits tumor cell proliferation in culture and colony formation in soft agar. This growth inhibition is partly the result of apoptosis induced by *MEG3* (Zhou et al., 2013). MEG3 induces accumulation of P53 protein and selectively regulates P53 target genes expression.

## Current Clinical Applications of hESC and hiPSCs

**FIGURE 4:**
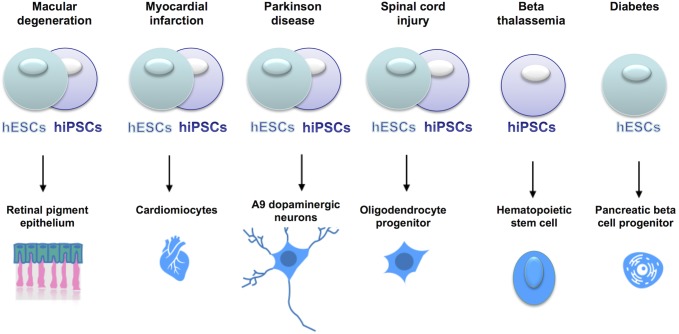
Current clinical trials using hESCs or hiPSCs for cell replacement therapies. In the online database www.clinicaltrials.gov, the clinical trials using stem cells are listed and described with details of the clinical phase, the type of cells used, the number of patients enrolled etc. Stem cells are currently tested in clinical trials to treat a series of clinical conditions, with some of them already at clinical phase IV.

Cell replacement therapies, whereby somatic cells of interest are generated from pluripotent stem cells, are currently under clinical trials (see Figure 4), for conditions such as macular degeneration and Parkinson’s Disease (Trounson and DeWitt, 2016; Guhr et al., 2018^1^).

We believe that hiPSCs showing imprinting defects should not be used for cell replacement therapies. For instance, *MEG3* LOI is linked to skeletal abnormalities [see section Uniparental Disomy 14 (UPD14)], thus hiPSCs displaying altered *MEG3* expression should not be used for the derivation of mesenchymal stem cells, that are progenitors of osteocytes. Similarly, because of the implication of LOI in cancer, hiPSCs with alteration of the *DLK1* DMR should not be used for the derivation of dopaminergic neurons or oligodendrocytes (*DLK1* LOI is linked to neuroblastomas and gliomas) or cells showing LOI at the IC1 DMR (*H19-IGF2*) should not be used for generating pancreatic cells (*IGF2* LOI is associated to pancreatic cancer).

A screening on the hiPSCs population, first for misregulated expression of imprinted transcripts, possibly followed by analyses of genetic mutations and epimutations, should be performed when choosing hiPSCs for cell replacement therapies. Additional safety measures could be considered, as in the case of the most advanced clinincal trials based on hESC-derived pancreatic beta cells, whereby cells are encapsulated before transplantation, in order to protect the host from potential harmful features of the cells.

## Strategies for the Detection of Correct Imprinting in hiPSCs

Loss of imprinting has been often measured in terms of loss of DNA methylation, but this does not always result in a loss of monoallelic expression, because other mechanisms (e.g., histone repressive marks) besides DNA methylation can regulate the expression of the silenced allele (Rugg-Gunn et al., 2007; Frost et al., 2014; Inoue et al., 2017). Furthermore, a decrease in the DNA methylation levels in hiPSCs would be of little to no consequence as long as the ZFP57-binding site remains methylated and targeted by the ZFP57/TRIM28 repressing complex (Zuo et al., 2012).

**FIGURE 5:**
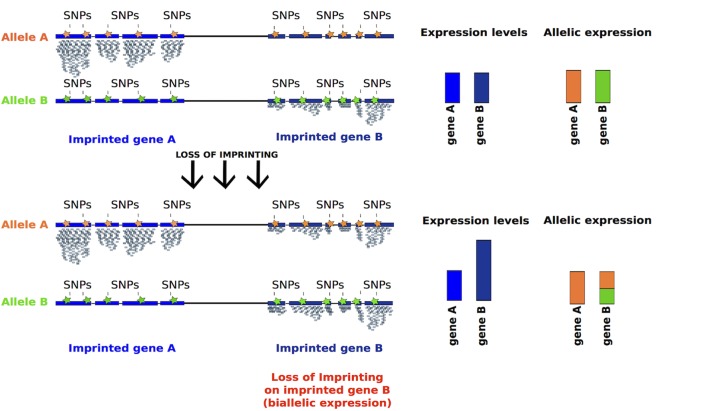
Inferring LOI from absolute expression levels and allelic expression derived from RNA-seq data. Reads mapping to either of the alleles can be discriminated using SNPs, represented in orange and green. Imprinted genes should be expressed monoallelically, thus SNPs associated to one allele should be detected. After LOI, reads containing SNPs associated to both alleles should be detected, as for gene B (bar half green and half orange). Moreover, the absolute expression of a gene should increase after LOI (taller blue bar).

Given that appropriate differentiation and proliferation of hiPSCs could be affected by the dose of imprinted gene transcripts, an effective way to measure LOI would be the measurement of levels of expression and the distinction of the mono- versus bi-allelic expression (Figure 5).

In particular, the two following analyses should be performed:

(1) Measuring the expression levels of imprinted genes, relative to a panel of control cells where imprinting is stable. This type of analysis can be easily applied to novel hiPSCs generated either by quantitative RT-PCR or by RNA-sequencing.
(2) Detection of monoallelic vs. biallelic expression based on SNPs (Santoni et al., 2017). For this analysis RNA-sequencing data should be analyzed to detect existing SNPs on imprinted transcripts, assigning reads originating from each of the two alleles (Figure 5). The limitation to this type of analysis is that some transcripts may not have informative SNPs.

## Conclusion

Loss of imprinting is observed in somatic cells in culture and, more frequently, in iPSCs, indicating that both the *in vitro* expansion and the reprogramming process contribute to LOI. Moreover, hiPSCs in a naïve state of pluripotency display more profound alterations at the level of methylation of imprinting control regions, while additional analyses will be needed to gauge the impact on the expression of imprinted genes.

Additional studies will be also needed to optimize culture conditions and reprogramming protocols in order to generate hiPSCs with more stable imprinting. At the same time such studies might shed light on the mechanisms of imprinting maintenance. For example, maternal and paternal imprints appear specifically more stable in primed and naïve hiPSCs (Bar et al., 2017; Giulitti et al., 2019) respectively, but the mechanisms underlying such differential sensitivity are unknown. Finally, given the potential detrimental effects of LOI, it would be important to develop standardized procedures for detection of altered expression levels and loss of monoallelic expression of imprinted transcripts.

In conclusion, hiPSCs represent an ideal tool for the study of regulation of genomic imprinting and, at the same time, a better understanding of the mechanisms controlling imprinted gene expression will have an impact on safety of hiPSC applications.

## Data Availability

No datasets were generated or analyzed for this study.

## Author Contributions

All authors listed have made a substantial, direct and intellectual contribution to the work, and approved it for publication.

## Conflict of Interest Statement

The authors declare that the research was conducted in the absence of any commercial or financial relationships that could be construed as a potential conflict of interest.

## Footnotes

1 https://clinicaltrials.gov
